# The Position of the Hand in Fracture of the Fore-Arm

**Published:** 1864-12

**Authors:** Paul F. Eve

**Affiliations:** Augusta, Ga.


ART. IV.—*The Position of the Hand in Fracture of the Fore-arm.* By PAUL F. EVE, M. D, Augusta, Ga.

I propose to call attention to the *mal*-position of the hand allowed by many surgeons of our army in treating fractures of the fore-arm. I am sure I have seen in Atlanta and Augusta hundreds of cases, within the past three years, in which the usefulness of the limb has been greatly impaired simply from the improper position of the fore-arm when broken. During this period patients have been examined, and wounded soldiers met in the streets, carrying the hand of the fractured member in a state of pronation, from which cause alone, when the bones will have re-united, the opposite motion, supination, will ever afterwards be rendered impossible. Men may be seen every day on the trains, by the wayside, in towns and villages, at home, even in hospitals, with the hands of broken fore-arms turned downwards and supported on boards, gutters and various kinds of splints, suspended from the neck by all sorts of slings; and most of them, no doubt, having these useful members permanently injured—losing forever the *all-important rotatory motion* of the radius upon the ulna, and consequently the ability to turn the hand over upon itself, or on its back. Now, if these are facts, and this loss of motion could have been prevented, and this too by the usual treatment recommended and pursued by the profession, then surely a grievous error, to say the least of it, has been permitted to obtain among our wounded.

As soon as I observed this practice, which has already, I apprehend, maimed for life thousands of our soldiers, I promptly and officially notified the Surgeon-General of the evil through the post-surgeon of Atlanta.

The proper position of the hand in fracture of the fore-arm is that which places the injured member between the states of supination and pronation. The limb should then be flexed at right angles, across the chest of the patient, with the thumb pointing directly upwards towards the chin. The main indication in the treatment is to preserve the interosseous space between the two bones of the fore-arm, upon the existence of which most important movements of the hand depend. It is true that in complete supination the radius is more widely separated from the ulna, but this being a forced position, prevents relaxation of muscles, and would be too painful to be borne; and it is certainly as true that its opposite or antagonistic position, pronation, by rolling these bones together, destroys all the space between them. Hence, with great unanimity, authors and practitioners treat fractures of the forearm on the principle described, keeping the limb thus confined some forty to sixty days. Fortunately, too, this treatment is applicable alike to one or both bones, or to whichever one may be broken. The usual apparatus, consisting of two rollers two and a half inches wide by six yards long; two light, straight splints, wide as the fore-arm to be treated, and in length extending from the elbow to the tips of the fingers; and two compresses, the size of the splints—are easy of access, answer a good purpose, and is an excellent substitute for many peculiar splints, &c., invented but not now attainable. With two additional compresses, three inches square and a half inch thick, when the radius is broken near the wrist, one placed over the lower end of the two bones, anterior surface, (hand supinated or nearly so,) and the other on the dorsum of the hand, I have managed for thirty-six years the various fractures of the fore arm, and I think with good results. The all-important indication in these cases is to have the anterior splint so long that the hand, during treatment, cannot become prone, that we may obviate the very condition now complained of, to be met with and deplored every day in our army.

